# Lay health workers perceptions of an anemia control intervention in Karnataka, India: a qualitative study

**DOI:** 10.1186/s12889-017-4758-x

**Published:** 2017-09-18

**Authors:** Arun S. Shet, Abha Rao, Paul Jebaraj, Maya Mascarenhas, Merrick Zwarenstein, Maria Rosaria Galanti, Salla Atkins

**Affiliations:** 1 Hematology Research Division, St. Johns Research Institute, St. Johns National Academy of Health Sciences, Bangalore, 560034 India; 2 Department of Hematology/Medical Oncology, St. Johns Medical College and Hospital, Bangalore, India; 3 Department of Family Medicine, Schulich School of Medicine and Dentistry, Western University, London, ON Canada; 4 MYRADA, Bangalore, India; 5 Department of Public Health Sciences, Karolinska Institutet, Stockholm, Sweden; 6 Centre for Epidemiology and Community Medicine, Stockholm Health Care District, Stockholm, Sweden

**Keywords:** Lay health workers, Complex community intervention, Pragmatic RCT, Implementation, Qualitative research, Focus group discussion, Childhood anemia

## Abstract

**Background:**

Lay health workers (LHWs) are increasingly used to complement health services internationally. Their perceptions of the interventions they implement and their experiences in delivering community based interventions in India have been infrequently studied. We developed a novel LHW led intervention to improve anemia cure rates in rural community dwelling children attending village day care centers in South India. Since the intervention is delivered by the village day care center LHW, we sought to understand participating LHWs’ acceptance of and perspectives regarding the intervention, particularly in relation to factors affecting daily implementation.

**Methods:**

We conducted a qualitative study alongside a cluster randomized controlled trial evaluating a complex community intervention for childhood anemia control in Karnataka, South India. Focus group discussions (FGDs) were conducted with trained LHWs assigned to deliver the educational intervention. These were complemented by non-participant observations of LHWs delivering the intervention. Transcripts of the FGDs were translated and analyzed using the framework analysis method.

**Results:**

Several factors made the intervention acceptable to the LHWs and facilitated its implementation including pre-implementation training modules, intervention simplicity, and ability to incorporate the intervention into the routine work schedule. LHWs felt that the intervention impacted negatively on their preexisting workload. Fluctuating relationships with mothers weakened the LHWs position as providers of the intervention and hampered efficient implementation, despite the LHWs’ highly valued position in the community. Modifiable barriers to the successful implementation of this intervention were seen at two levels. At a broader contextual level, hindering factors included the LHW being overburdened, inadequately reimbursed, and receiving insufficient employer support. At the health system level, lack of streamlining of LHW duties, inability of LHWs to diagnose anemia and temporary shortfalls in the availability of iron supplements constituted potentially modifiable barriers.

**Conclusion:**

This qualitative study identified some of the practical challenges as experienced by LHWs while delivering a community health intervention in India. Methodologically, it highlights the value of qualitative research in understanding implementation of complex community interventions. On the contextual level, the results indicate that efficient delivery of community interventions will require streamlining of LHW workloads and improved health system infrastructure support.

**Trial registration:**

This trial was registered with ISRCTN.com (identifier: ISRCTN68413407) on 23 September 2013.

## Background

Lay health workers (LHWs) are frequently used to alleviate human healthcare resource shortages in a variety of settings [1, 2]. They deliver a myriad of health interventions, including those directed at maternal and child health, chronic infectious diseases (e.g. tuberculosis and HIV) and immunization uptake [3]. One of the key areas in relation to LHW use as interventionists is their performance, which in turn is closely related to their perceptions of the intervention at hand [4, 5]; and these perceptions can influence a number of facets of intervention implementation including delivery and end user uptake [6]. As LHW performance is pivotal to program implementation, it is important to study their thoughts and experiences when implementing effectiveness trials designed to inform wider health system intervention roll-out [7, 8]. LHWs deliver several community health programs in India [1, 6, 9, 10], yet few qualitative research studies have explored implementation aspects that may influence the outcomes of such programs [11, 12] and none have been conducted in India. In particular, these factors need to be studied and understood, considering that the primary health care system in India heavily rests on LHW support [10, 13].

Anemia, an indicator of nutritional health is prevalent among Indian children. Successful public health control of anemia through the national nutritional anemia control program has previously been hampered by inadequate programmatic implementation [14]. We evaluated a novel LHW-led intervention delivered in the setting of the integrated child development scheme (ICDS) in rural province of Karnataka state in South India [15]. A qualitative study was embedded in this trial, aiming primarily at understanding LHWs’ acceptance of the proposed intervention and factors affecting its implementation [7] to explain trial results when available. This kind of information is often of great value in guiding the scaling up of effective interventions [16, 17]. We also sought to appreciate the political, socioeconomic and cultural conditions of the LHWs working environment, since this would be relevant to broader context of the intervention and its subsequent scale up.

### The cluster randomized controlled trial

#### Context

Chamarajnagar district, in Karnataka, South India, presents a favourable profile of population health (fertility rate, immunization coverage, antenatal care, infant mortality rate, institutional delivery), occasionally exceeding goals set by the National Rural Health Mission (NRHM) [18, 19]. However, in spite of good primary health care indicators, rates of anemia in children and women are high in the district, closely mirroring the national average [18–20]. The trial tests the hypothesis that a novel contextually relevant LHW led intervention delivered to mothers of anemic children would result in improved anemia cure rates compared with treatment as usual.

#### ICDS, LHW duties and work environment

The ICDS, a federally administered scheme of the NRHM, provides early child nutrition and preschool non-formal education through a network of village based anganwadi day-care centers (ADCs) [21]. A frontline anganwadi worker, henceforth referred to as LHW, manages the ADC and forms an integral component of the village health system by improving the nutrition and health of 0–6 year old children. As a contracted non formal employee of the ICDS, the LHW is directly supervised by the ICDS Child Development Program Officer (CDPO) but also reports to local education/health officials of the Karnataka state government. Typically, this LHW is a literate educated female sourced from the village and respected by the community. She maintains childhood immunization records and conducts health education for mothers alongside her daily kindergarten education activities. She also administers and distributes a monthly ration of food (nutrition supplement), evaluates village children for anemia, and distributes iron supplement tablets in accordance with the National Iron + initiative [22].

#### The intervention

We hypothesized that educating mothers about anemia, nutrition, IFA supplementation, and hygiene would lead to a perception that their actions could control anemia in their children. Informed by the social cognitive theory (SCT), three main factors affect the likelihood of health behavior modification: 1) self efficacy, 2) goals, and 3) outcome expectancies [23]. Training imparted by the LHWs to mothers of anemic children sought to increase the mothers knowledge, and address all three factors by promoting expectations about their children’s health outcomes, improving self-efficacy by facilitating their learning and reinforcing their positive behaviors, eventually leading to reduction in childhood anemia [15]. The specific activities of the LHW were to: i) educate mothers and improve their awareness with respect to anemia, ii) counsel mothers about a contextually relevant iron rich diet and promote dietary diversification, and iii) monitor and improve adherence to supplementary iron and folic acid (IFA) in children. We assessed the LHWs’ perceptions and experiences of this intervention.

### The qualitative study

#### Participants

The LHWs involved in the trial were literate, had at least primary education (most had secondary), and were usually residents in the village for which they were in charge of children’s care (Table 1). Attempting to obtain views of both well and less well performing LHWs in the trial before the trial had started, we used performance during training as indicative of probable success in intervention implementation. Therefore, from 30 LHWs that participated in the intervention training, we purposively sampled well performing (*n* = 5) and less well performing (n = 5) LHWs based on a questionnaire used to evaluate training success. Subsequently, two LHWs implementing the intervention easily and two LHWs encountering difficulty implementing the intervention as assessed by the trial research team, were purposively selected and observed in a non-participant context over multiple time points during the intervention to obtain a greater understanding of the LHW working environment. The observations assessed LHW interactions with children and mothers, counselling and education sessions when conducted, and use of the supplementary educational material. Daily LHW activities in her working environment including distribution of nutrition supplements and recording of data were also observed.

**Table 1:** Participant demographic information

Village	LHW No.	Age	Education (years of education)	No. of 12–59 month children in village
FGD #1	1	42	12	49
	2	35	9	11
	3	59	10	45
	4	42	10	44
	5	50	10	38
	6	38	10	45
	7	45	10	55
	8	54	15	43
	9	49	10	61
FGD #2	1	35	9	11
	2	42	10	44
	3	50	10	38
	4	38	10	45
	5	42	12	49
FGD #3	1	45	10	55
	2	54	15	43
	3	49	10	61
	4	26	10	19
	5	55	10	20
Observations	1	45	12	58
	2	46	12	61
	3	47	10	60
	4	50	10	38

*LHW* lay health worker, *FGD* focus group discussion

## Methods

### Data collection

Our data collection was conducted iteratively, concurrently with data analysis. Focus group discussions (FGDs) were followed by non-participant observation. Field notes complemented the data collection and analysis process.

#### Focus group discussions

The FGDs were conducted in the local language by experienced researchers working in the main study. The first FGD (FGD 1) was conducted immediately after the final training session and focused more on training for the intervention. Nine of the ten LHWs agreed to participate. Subsequently, during intervention delivery, two additional FGDs (FGD 2 & 3) focusing on intervention delivery were conducted with the same group of LHWs. One of the nine LHWs was unable to continue participating in the remaining FGDs (FGD 2 & 3). This resulted in the inclusion of two additional LHWs (one well performing and one less well performing) for FGDs 2 and 3 (total LHWs invited to participate = 12). The same topic guide was used for all groups and included general LHW duties, barriers and facilitators to their work, relationships with mothers in the community, and their perceptions regarding training for the intervention, the intervention itself, and its implementation. FGDs were audiorecorded, transcribed and translated into English using a professional translator, checked for accuracy by AS and AR, both fluent in English and Kannada, the original language. Transcriptions were then independently coded by AR (social scientist) and AS (medical doctor).

#### Non-participant observations

When the trial was mid way through completion, non participant observations were conducted with LHWs delivering the intervention by a research team member. The research team selected 4 from the 30 LHWs to conduct observations with, based on their performance and location (one of these LHWs was a participant in the FGDs). The observations occurred in the setting of the natural work environment of the LHW.

#### Field notes

A member of the research team also maintained detailed field notes during the training sessions, FGDs, and the nonparticipant observations. These were used as supplementary material and provided contextual and in-depth information on emerging themes.

### Analysis

We used the framework method for analyzing data to ensure systematic analysis of themes identified across different sources of transcripts [24–27]. The analysis of FGDs focused on LHW perceptions, acceptability and experiences with regard to the intervention particularly focusing on barriers and facilitators to its implementation [5]. Transcripts of the data corpus were read and re-read to gain familiarity with the data so that recurring ideas could be identified. At this point, each researcher in the team recorded interesting issues and made notes of issues identified and potential themes from the data. The team met to discuss these. After this, each transcript was coded manually, and from these codes, abstractions were developed to form categories by inductive reasoning. From these categories, codes were built up into subthemes and themes in order to develop a coding framework. Each researcher used this coding framework independently to assign data to the themes and categories in the coding matrix using Excel (Table 2) [25]. After coding, themes and categories were discussed among the research team, any disagreements were resolved by discussion. The coding process was conducted within and across LHW groups, and across different sources of data, actively paying attention to differences and similarities. Participant observations and field notes were similarly manually coded and analysed inductively (Table 3).

**Table 2:** Example of the initial analytic process

Meaning unit	Code	Subtheme	Theme
‘We can’t tell too many things to them. We tell them slightly little more if we feel like they listen. If they show negligence or lethargy, we don’t bother too much about them. We tell what needs to be told and return. Some show interest, others may have less interest slightly because of their busy work or visiting some place or in a hurry. It is possible that they can’t give attention.’	Challenging to retain mothers interest and attention	Variable mother LHW relationship	Interpersonal relationship with mothers

*LHW* lay health worker

**Table 3:** Example of use of the observation notes as supportive material

Theme	Working conditions challenging intervention	
Subtheme	Position is one of responsibility, which they take seriously	Duties are extensive and varied; wide ranging tasks
FGD1	We have come here to do it, and now we will do it, how much ever the difficulty.	
FGD2		[Long discussion about the various logs, registers an diaries they maintain, involving maternal, child, and community health; registrations of births, deaths, and everything in between; educational responsibilities; official records; and account and book keeping]
FGD3		Same comment as above, re: completion of registers, logs, and diaries
Field notes	LHWs clearly view their position and work with pride: this is evident in the way they dress and authoritative way they carry themselves. Most take their responsibilities seriously, with the intent of fulfilling them.	On a daily or weekly basis, LHWs perform, at a minimum: nonformal preschool activities, ration distribution, 1–2 meetings related to maternal and child health or related issues, home visits, meetings with or visits by supervisors, and completion of various registers.

*LHW* lay health worker, *FGD* focus group discussion

### Ethics and consent to participate statement

The study sought and obtained approval from the St. Johns National Academy of Health Sciences Institutional Ethical Committee (IEC 115/2012). We obtained written informed consent from each participating LHW. The LHWs were not offered inducements to participate in the qualitative studies.

## Results

All participants were actively involved in the FGDs. In our analysis, we identified the following major themes: i) LHWs initial acceptance of the intervention changing over time; ii) LHWs innovating and adapting the implementation protocol; iii) precarious relationships with mothers in participating communities; and iv) working conditions challenging intervention delivery.

### LHWs initial acceptance of the intervention changing over time

All LHWs felt that the intervention was acceptable and feasible to deliver during their routine activities, and most appeared genuinely pleased to participate in a trial seeking to improve children’s health. However, optimistic sentiments about the actual implementation of the study, such as the following, were few:

*‘Now that we are giving tablets and informing them about food, mothers can also understand... This makes both mothers and us happy. We will also get happy because we can improve children a bit’ (FGD #3, LHW 1)*

In the initial FGD, LHWs suggested ways to continue intervention work even after the trial was complete. However, such positive sentiments diminished in subsequent FGDs, as the trial progressed. Moreover, since the research team conducted point of care blood testing to detect anemia in children and actively sourced IFA supplies during supply shortfalls, LHWs expressed doubt about their ability to continue such activities after the trial was complete:

*‘We can give them information regularly. But what about tablets? Who gives all that? We explain them about food and nutrition benefits... If there is anaemia child what we do? We don’t have the facility to take care them... Blood test is also a big problem. How can we check a child of having anaemia?’ (FGD #2, LHW 8)*

*‘We can say about diet. We can tell children to take something that is available at home. But about the tablet, we can provide only if you can provide it to us. Otherwise [we cannot continue]... And about testing, you have to conduct a blood test. Otherwise no.’ (FGD #3, LHW 1)*

The waning of enthusiasm for the intervention from the first FGD to subsequent ones was also seen through comments on the number of forms the intervention required and confusion about how to correctly fill these. In a later FGD, LHWs described the forms as a burden but later after familiarization with this activity some found it more acceptable:

*‘We were unsure of how to work with these, this new additional burden. Already we had many books to write and now we were given a new one... we thought in the beginning [it is a burden], but now it’s nothing’ (FGD #2, LHW 8)*

Initially, some LHWs thought that completing the data collection instruments was a relatively simple task but subsequently developed confusion about expectations. These LHWs expressed the following lack of clarity:

*‘Should we write all the dates if we visit them 2-3 times in a day? ... Now we went for counselling. Then we visit them within a week. We visit them 5-6 times in a month. Is one date enough during those occasions?’ (FGD #3, LHW 1)*

### LHWs innovating and adapting the implementation protocol

The discussion regarding how to implement the intervention was rich and indicated how LHWs had modified the intervention to suit their daily tasks. Most LHWs carried out the intervention along with their other duties such as home visits, or ration distribution:

*‘[We do the counselling] when we are together, when we distribute rations. We tell them when all are together.’ (FGD #3, LHW 1)*

*‘Sometimes they did not come even when we call them. Sometimes we do not get opportunities to meet them even when we visit. Then we meet them at the ration. This meeting is inevitable for them, where we explain this thing.’ (FGD #3)*

This finding was corroborated by non-participant observations, which showed how LHWs found ways to incorporate the intervention tasks into their regular work. In addition, we observed that most LHWs did not use the supplementary material to help facilitate their education and counselling sessions but depended on their memory.

### Precarious relationships with mothers in participating communities

The successful implementation of the intervention depended on the LHWs interacting with the mother of the anemic child to impart specific education, nutritional information, deliver IFA supplements and monitor adherence. However, relationships between the mothers and the LHWs were precarious and even fickle, as LHWs reported mothers’ disengagement and disinterest in these meetings:

*‘They [the mothers] ask us to make it fast. They keep [checking] the time every hour. They have to work with their cows and calves, etc.’ ‘... they do not want to learn. They don’t have much patience for these things.’ (FGD #2, LHWs 3 & 8)*

The perceived inattention or lack of interest on the part of the mothers was a source of frustration for LHWs. They discussed the mothers forgetting or ignoring instructions at length:

*‘We can’t tell too many things to them. We tell them slightly little more if we feel like they listen. If they show negligence or lethargy, we don’t bother too much about them. We tell what needs to be told and return. Some show interest, others may have less interest slightly because of their busy work or visiting some place or in a hurry. It is possible that they can’t give attention’ (FGD #3, LHW 10)*

#### Multiple roles complicating the relationship

Another factor that complicated the relationship between LHWs and mothers was that LHWs served the community in various roles, such as being leaders or enforcers of village micro credit initiatives:

*‘[Relationship with mothers] is generally good. But sometimes if we ask them to pay the loan back then there is some misunderstanding. Later they may realize we tell them for their own good and they are fine.’ (FGD #1, LHW 8)*

LHWs play a multifaceted role in the community that possibly requires them to draw on their interpersonal skills, that could challenge uniform implementation of an intervention in the setting of a trial. The LHW-mother relationship seemed to evolve across time and possibly other contextual issues, such as religious backgrounds. However, the LHW-mother relationship seemed to also have potential for conflict particularly based on the expectations of mothers:

*‘It is possible they appreciate you when you do good things; it is possible that they blame you when you fail.’ (FGD #2, LHW 6)*

#### Status in the community as positively influencing mother-LHW relationships

The mother-LHW relationship was also closely related to the LHWs overall status in the community. LHWs identified meeting with mothers to educate them about child health and good nutrition practices as satisfying.

The LHW was familiar with her role, and her status and pride of position in the village was evidenced by the manner in which villagers greeted her:

*‘We feel like each house is like our house... we talk to the people while going around and we become familiar with the people.’ (FGD #1, LHW 6)*

LHWs also took pride in their work and perceived themselves as valuable contributors to the village community. This finding was supported by our field observation notes, where the researchers noted that the LHWs usually dressed in official government issued uniforms and handled distribution of nutrition rations to villagers with diligence and responsibility (Table 3). They seemed invested in the well being of the mothers and their children.

All LHWs found that the work of caring for children attending the ADC was satisfying and enjoyable:

*‘When we see the children we feel happy’ (FDG #1, LHW 9)*

*‘If there is a problem at home, when we see the children we forget it.’ (FGD #1, LHW 1)*

One LHW felt that mothers appreciated them:

*‘When they get to know they are pregnant, they run towards us!’ (FGD #2, LHW 6).*

### Working conditions challenging intervention delivery

#### Wide ranging services and high work burden

ADC LHWs primarily have health-related duties, but are also responsible for a variety of educational (e.g., teaching pre-school) and administrative (e.g., maintaining voter records) duties in their village. They also maintain extensive and updated records related to their activity and attend regular meetings and health related training sessions. With this background, LHWs seemed to speak of their perceptions of the intervention related workload with resignation in the initial group:

*‘We have come here to do it, and now we will do it, however much the difficulty’*

*‘We will do whatever needs to be done... we have come to that level’ (FGD #1, LHW 6)*

However, this perception seemed to change and in the later FGDs, LHWs did not appear to find the additional intervention related activities particularly burdensome since they reported completing tasks within their regular work hours. This was also confirmed during the non-participant observations. It is possible that LHWs found additional intervention activities that they could complete during the regular working day hours burdensome since it was not remunerated.

#### Salary concerns

Combined with their perceptions of a heavy workload, LHWs also felt a lack of recognition by their employer (state and national government) and adequate remuneration, which led to considerable dissatisfaction:

*‘We are working more and getting less salary... we are not satisfied. If we get at least Rs. 10,000 [about twice what they currently earn], we would be happy.’*

*‘Our salaries are low. We don't get salaries proportionate to our work’ (FGD#2, LHW 3)*

#### Job security concerns

Privatization of the services they provided was another concern, which appeared to make their continued employment less secure:

*‘They have decided to privatise the whole sector. Privatisation means they give ADC responsibilities to a private institution. They planned to give whole authority to Coca-Cola company. There is a big difference when responsibility is shifted to the private institution from the government. We may not get salary promptly. They may give more work stress to us. We may attend without missing a day. We may not get casual leaves properly. We may face many problems. They... may expel any worker whenever they want.’ (FGD #2, LHW 8)*

These two obstacles prompted LHWs and their unions to make demands for improved salaries and benefits (salary protection, pensions, subsidized healthcare). As indicated in the field notes, a 3-week Karnataka state wide strike by the LHWs brought all trial related activities to a complete halt. During this period the research team stopped all research activity and paid heed to the LHW concerns.

#### Inadequate employer support and recognition

Financial remuneration was not the only source of dissatisfaction among LHWs in terms of employer support. At the local level, village councils (*Gram Panchayats*) and their leaders played a significant instrumental role. Some LHWs reported that councils were either extremely engaged (for example: painting walls, building/fixing toilets, and providing supplies) or not at all:

*‘We are asking them from the past four years to close the drainage pit, but we didn’t get any help from them’ (FGD #3, LHW 1)*

*‘They will put the signature, but they will not help us’ (FGD #1, LHW 3)*

The range in *Gram Panchayat* engagement was noted in our non-participant observations and field notes; in some villages, leaders stopped by to meet with the field research team or chat with the LHW, but in others, there was little or no communication. In the former, village leader involvement seemed to result from the LHW being highly engaged with the community with respect to the intervention. At the district supervisor level, LHWs sought a sympathetic understanding of their working conditions. One LHW complained about her difficulties in travelling for work:

*‘Sometimes I cry, for my legs hurt from walking that distance... Sometimes bus, auto, and bicycle also increase my body pain*... *I applied for a transfer many times, but no one listens or reads my request letters’ (FGD #2, LHW 8)*

Notably, several LHWs praised a retired district-level supervisor who had demonstrated compassion in terms of accommodating the needs of LHWs who were pregnant and required medical assistance.

## Discussion

LHW roles have evolved with increasing emphasis on community based health care particularly in countries with robust primary care programs [3, 28]. A recent systematic review categorized factors influencing LHW performance in low middle-income countries into three categories: i) broad contextual factors, ii) health system factors, and iii) intervention design factors [4]. Our study identified factors influencing implementation of an iron supplementation intervention by the frontline LHW in the setting of the integrated child development scheme in rural Karnataka India. In particular, we found several modifiable aspects of the proposed intervention that could positively affect LHW implementation of the intervention including: a) documentation load, b) requirement to perform home visits to deliver the intervention, and c) complexity of documentation. We also identified contextual level factors: a) excessive work burden, b) poor LHW motivation and c) too wide a range and too many LHW tasks, and health system factors: a) limited ability to detect anemia, b) unreliable IFA supplies, c) inadequate public remuneration, and d) concerns related to privatization of the ICDS, that would potentially influence intervention delivery. These findings may be applicable both to other states in India and to other LHW led interventions addressing communities from a health or socioeconomic perspective [6, 29].

Experience shows that LHWs recruited from local communities have a greater impact on resource utilization, creation of health awareness and overall health outcomes [2, 30]. Despite this apparent strength, we found that frontline LHWs delivering our intervention in the ICDS context faced challenges due to their burden of work, poor remuneration, and wide ranging nature of tasks. Overburdening of LHWs would result in less optimal implementation of interventions such as ours, more so in villages densely populated with children where even more clients require services. Moreover, provision of a wide range of mostly preventive not curative services by the LHW may reduce the village community’s confidence in the their overall effectiveness [31], explaining at least in part the ambivalence of mothers to some of the LHWs delivering this intervention. Primary health worker credibility is also linked to their ability to provide curative services and an adequate supply of drugs [32], thus efforts to support the LHW mother relationship from this perspective would aid successful implementation of this intervention.

Participant LHWs expressed the desire to conduct their daily activities as well as the intervention but were clearly frustrated by their work burdens, terms of public remuneration and employer support. LHWs thought the intervention posed an additional burden despite completing their intervention activities during regular hours because they performed the extra activities without any additional reimbursement. Providing incentives to LHWs could potentially help change such attitudes but sustaining incentive based performance improvements over the long term will prove to be challenging [33]. LHW demotivation due to inadequate remuneration seemed further fuelled by fears of possible privatization of the ICDS. The trend of outsourcing primary health care services by the Karnataka government to private public partnerships has possibly led to some of these concerns [34, 35]. These remuneration and privatisation concerns were serious enough for LHWs and their unions to act by repeatedly calling for strikes, resulting in a 3-week Karnataka state wide strike to collectively bargain for increased wages, formalized employment status, and stoppage of privatization efforts. Insufficient reimbursement and lack of performance based incentives for LHWs will challenge successful implementation of the intervention in this context.

The study identified two major ways in which LHWs modified intervention delivery to address their workload related concerns. First, LHWs incorporated intervention associated education and counselling activity into their routine work rather than make separate visits to the homes of mothers of anemic children. Second, most LHWs did not refer to the educational support material although this could have reflected familiarity with the intervention messages. It is quite commonplace in implementation for the interventionists to adapt/change the intervention and how it is supposed to be implemented [36, 37]. Delivering the intervention differently from the method observed during training while maintaining the key information imparted during the training demonstrated LHWs capacity to innovate and adapt the intervention to their regular working environment. However, from a trial perspective, such modifications while unavoidable in a pragmatic trial affect the fidelity to the actual intervention protocol and potentially influence trial results.

This qualitative study has provided insight into factors affecting LHWs implementation of interventions and if successful, and can contribute to strengthening iron supplementation programs aiming to reduce anemia prevalence. However, further insight would be needed in particular to the experiences of the mothers of children participating in the intervention. The findings also need to be considered in the context of the following limitations. First, the study was conducted in the context of a randomized trial, which may generate responses that are not typical of LHWs who do not participate. Second, the number of FGDs performed was relatively small raising concerns regarding the validity of the findings. Finally, generalizability of these findings is not possible. Among the strengths was the use of two independent analysts and data triangulation, which enhanced the validity of the results. Moreover, the FGDs provided a deeper understanding of the LHW working environment in Karnataka and of the broader socio-political context of anganwadi workers in India.

## Conclusion

This study identified LHW acceptance and perceptions of a novel anemia control intervention and health system level barriers that could hamper the implementation of this particular intervention. The results provide useful evidence of the importance of contextual factors in influencing the effectiveness of a novel community health worker led intervention seeking to improve child health outcomes in India. While the intervention was found acceptable to LHWs, several individual and health system barriers limiting optimal implementation were identified. Attempts to address these barriers identified on the part of LHWs and to strengthen the health system in this context could overall help facilitate optimal implementation of this complex community intervention. Disseminating these findings to policy makers could help secure economic resources and political commitment to improve the conditions of LHWs in this setting and permit effective delivery of community based maternal and child health interventions.

## Abbreviations

ADC: Anganwadi daycare center
FGD: Focus group discussion
ICDS: Integrated child development scheme
IFA: Iron and folic acid
LHW: Lay health worker
NRHM: National rural health mission
RCT: Randomized controlled trial
